# Validation of the German version of the state mindfulness scale for physical activity in a clinical sample

**DOI:** 10.1186/s41687-024-00815-8

**Published:** 2024-12-02

**Authors:** Jan Wallner, Leona Kind, Carolin Donath, Johannes Kornhuber, Katharina Luttenberger

**Affiliations:** 1 Department of Psychiatry and Psychotherapy, Centre for Health Services Research in Medicine, Friedrich-Alexander-Universität Erlangen-Nürnberg, Schwabachanlage 6, 91054 Erlangen, Germany; 2 Department of Psychiatry and Psychotherapy, Friedrich-Alexander-Universität Erlangen-Nürnberg, Schwabachanlage 6, 91054 Erlangen, Germany

**Keywords:** Mindfulness, State, Validation, Physical activity, Exercise, German

## Abstract

**Background:**

Mindfulness has been studied as a treatment option for a large range of psychological disorders and is associated with a multitude of positive psychological outcomes. There are now several scales for measuring mindfulness as both a trait and a state. As mindfulness potentially plays a critical role in maintaining physical activity habits, the State Mindfulness Scale for Physical Activity (SMS-PA) was developed to specifically measure mindfulness in a physical activity context. This study aimed to provide validity evidence for a German version of the SMS-PA (henceforth called SMS-PA-G) in a clinical sample.

**Methods:**

We used baseline data from 129 participants in the randomised controlled trial ‘New Ways to Cope with Depression’. The sample, which was screened for clinical depression symptoms, completed the SMS-PA-G and several other psychometric scales for capturing state mindfulness, self-efficacy, sense of coherence, anxiety, depression, and physical activity. We conducted reliability and item analyses and ran a confirmatory factor analysis. Also, we assessed construct validity through correlations with the abovementioned scales and through differences in SMS-PA scores between physically active and nonactive participants.

**Results:**

The mean SMS-PA-G score in our sample was 25.3 with a standard deviation of 8.5. The item and reliability analyses provided satisfactory Cronbach’s alpha and discriminatory power values. The confirmatory factor analysis showed that physical activity mindfulness can best be described via a bifactor model, with specific mind and body factors and a general mindfulness factor. We found the expected relationships with the attention subscale of state mindfulness, self-efficacy, and sense of coherence but did not find them with the awareness subscale of state mindfulness, depression, and anxiety. As hypothesised, physically active participants exhibited higher SMS-PA-G values than nonactive participants.

**Conclusions:**

The SMS-PA-G is an internally consistent test instrument that captures respondents’ general physical activity mindfulness and their attention to mental and bodily events. Whereas validity evidence was generally supportive of the SMS-PA-G, its relationships with other constructs require further investigation.

**Trial registration:**

ISRCTN, ISRCTN12347878. Registered 28 March 2022, https://www.isrctn.com/ISRCTN12347878.

## Background

In the early 1980s, Jon Kabat-Zinn introduced mindfulness in a clinical context [1]. Kabat-Zinn drew heavily from various strands of Buddhist tradition and Indian philosophy to create a mindfulness programme to treat patients with chronic pain. As patients let their attention move freely from moment to moment without judgement, the perception of pain was decoupled from the affective response, and their suffering was reduced. Since this seminal application of the concept, mindfulness has been integrated into the treatment of many other disorders [2], such as depression [3], anxiety disorders [4], borderline personality disorder [5], addiction [6], and eating disorders [7]. Interest in mindfulness received an additional push when Brown and Ryan showed that mindfulness is associated with a wide range of well-being and mental health indicators, such as self-esteem, positive affect, life satisfaction, and lower levels of depression and anxiety [8]. Simultaneously, several challenges have arisen in mindfulness research. Often, mindfulness interventions cannot be compared due to a lack of detailed descriptions of the procedures; also, the actual conceptualisation and definition of mindfulness is subject to ongoing debates [9].

A relatively novel branch of mindfulness research is concerned with the study of mindfulness in the context of physical activity. Since regular physical activity can prevent or treat numerous physical health conditions, such as coronary heart disease, stroke, diabetes, colon cancer, and breast cancer, an increase in physical activity throughout the population is an important public health goal [10]. Similarly, there is an association between physical activity and mental health [11] and physical activity serves as a strategy in the promotion of mental health and in the prevention and treatment of mental disorders [12] such as depression [13–15], anxiety disorder [16, 17], and post-traumatic stress disorder [18]. Experiencing mindfulness during physical activity may increase the motivation to do sports and may thus propagate the positive effects of physical activity [19]. Conversely, there is empirical evidence that physical activity may increase mindfulness [20]. Self-determination theory (SDT)—which breaks motivation down into an intrinsic component, several extrinsic components, and the basic needs of autonomy, competence, and relatedness [21]—offers a model that explains how mindfulness can increase the motivation to do sports. It is plausible that carefree immersion in an activity and the perception of pleasant bodily sensations would increase intrinsic motivation, that is, the enjoyment of the activity per se [19, 22]. At the same time, this could lead to extrinsic motivators (e.g. concerns about looks or rewards from exercise) being less important. Indeed, there is already evidence that physical activity mindfulness is positively correlated with intrinsic motivation and negatively correlated with body surveillance (i.e., concerns about one’s appearance during exercise) [19, 23]. SDT asserts that such motivation coming from within is central to human agency and leads to increased enjoyment, persistence, and performance [24]. In a physical activity context, several studies have supported its association with increased frequency and duration of activity [25, 26] as well as habit strength [27].

Available mindfulness scales have traditionally measured the construct as either a trait or a state. The most popular of the numerous trait scales [28] are the Mindful Attention Awareness Scale (MAAS) [29], the Five Facet Mindfulness Questionnaire (FFMQ) [30], and the Kentucky Inventory of Mindfulness Skills (KIMS) [31]. There are considerable differences in how these questionnaires conceptualise mindfulness. For example, whereas the MAAS captures mindfulness solely as the opposite of an inattentive mental disposition, the FFMQ and KIMS postulate mindfulness as a multidimensional construct with dimensions such as *Observing*, *Acting With Awareness*, and *Nonreactivity*. The state scales, which include the Toronto Mindfulness Inventory (TMI) [32], the Multidimensional State Mindfulness Questionnaire (MSMQ) [33], and the State Mindfulness Scale (SMS) [34], are similarly heterogeneous. Whereas the SMS distinguishes between mindfulness of mind and mindfulness of body and focusses primarily on attention, the TMI’s dimensions (*Decentring* and *Curiosity*), as well as the MSMQ’s dimensions (*Acting With Awareness*, *Nonjudgment*, and *Attention*) have an added emphasis on a person’s attitude while observing. As a common thread, mindfulness as trait is seen as a general tendency to be mindful [8] whereas mindfulness as state describes the manner in which an activity is carried out [2]. In the latter view, mindfulness is seen as an active process that can be cultivated and lends itself to intervention [32]. State mindfulness may be measured during interventions, thus providing information about their efficacy [32]. Similarly, it may be measured during physical activity to investigate the link between present-moment attention and motivation and other constructs [35]. There are several arguments in favour of such measurement with a scale that is specific to the physical activity context [36]. First, certain items from available state scales are not appropriate for assessing mindfulness during physical activity (e.g. the first item from the MSMQ reads ‘I did tasks/things automatically without being aware of what I’m doing’) [33, p. 742]. Secondly, the behaviour emanating from a given trait typically varies to a great extent across situations [37, 38] and physical activity with the associated changes in physiological functions may be assumed to be a rather particular situation. Thirdly, evidence from psychological flexibility research shows that context-specific measurement performs better than more generic measurement [39]. And finally, the extant mindfulness research has mainly focused on the relationship between trait mindfulness and physical activity in general, but ‘there is an absence of evidence investigating the micro-temporal association in mindfulness and physical activity level’ [40, p. 14].

Hence, the State Mindfulness Scale for Physical Activity (SMS-PA) was developed from the SMS [34] to capture state mindfulness in a wide range of physical activity contexts. It adapted the items of the SMS to cover the full spectrum of bodily sensations experienced during exercise [19]. The SMS in turn had been developed as an alternative to the preexisting state scales MAAS [29] and TMI [32] due to concerns about content validity and applicability (e.g., the MAAS measures mindfulness only during daily activities; the TMI focusses primarily on mindfulness during meditation practice). The SMS was based on a consensus definition of mindfulness [2, p. 232] that attributes two central components to it: ‘attention (…) maintained on experience’ and a stance ‘characterized by curiosity, openness, and acceptance’. Building on this contemporary view, Tanay and Bernstein [34] further drew on traditional Buddhist texts to arrive at the two main domains towards which this attention can be directed: mental events (*Mind*) and physical sensations (*Body*). Like the SMS, the SMS-PA has a subscale that measures mindfulness towards thoughts and emotions and a second subscale that measures mindfulness towards bodily sensations. Sound and diverse validity evidence has been provided for both the original version of the SMS-PA [19] and its Spanish translation [22].

With this study, we aimed to provide validity evidence for a German version of the State Mindfulness Scale for Physical Activity (henceforth called the SMS-PA-G) and establish the first German scale for measuring mindfulness in the context of physical activity. We translated the existing English-language scale into German and performed internal consistency and item analyses. In addition, we (1) examined the SMS-PA-G’s factor structure, (2) tested its construct validity via correlations with theoretically related constructs, and (3) compared mindfulness levels among physically active and nonactive participants. On the basis of the SMS-PA’s original validation study [19], we expected to find a bi-factor structure. We assessed construct validity via correlations between physical activity mindfulness (SMS-PA-G) and mindfulness as a state (MSMQ) [33]. Given the SMS-PA-G’s focus on perception, we expected stronger correlations with the MSMQ subscales that emphasise attention and awareness. As mindfulness is typically associated with cognitions of optimism and competence [8], we computed correlations with Self-Efficacy and Sense of Coherence to further corroborate construct validity. Conversely, given that mindfulness is typically inversely related to measures of psychological burden [29], we correlated the SMS-PA-G with measures of Depressiveness and Anxiety. In line with results from previous validation studies of the SMS-PA [19, 22], we assumed that people become more in tune with themselves as they repeatedly engage in physical activities. Hence, we hypothesised that our physical activity mindfulness scale would return higher values for the group of regularly active participants.

## Methods

### Design

The data for the validation were gathered in the baseline assessment of the randomised controlled trial ‘New ways to cope with depression’ (ISRCTN 12347878). We investigated the effect of bouldering psychotherapy and mental model therapy on depression compared with a control group. Furthermore, we analysed various secondary outcomes, including additional psychometric variables, physiological indicators, and the impact factors of the therapies. Participants were recruited in the Erlangen-Nuremberg-Fuerth metropolitan region via informational materials, information given to health professionals, and several online and social media resources. They entered the trial in one of four intervention waves, which ran from May 2022 to May 2023. Each intervention period lasted for 10 weeks with weekly 2-hour therapy sessions for the participants in the treatment groups. Data were collected at baseline (t0), directly after the intervention (t1), and 12 months after the intervention (t2). The Ethics Commission of the University Hospital Erlangen approved the protocol of the study (date of approval: 13.12.2021; reference number: 21-332-B). A detailed description of the study design is available in a separate publication [41].

### Screening

A total of 170 potential participants were screened via online video interviews. Of these, 129 met the criteria and formed the intention to treat sample. Inclusion criteria were acute symptoms of depression, informed consent to participate in the trial, the ability to get to the therapy locations at the prescribed time, and access to electronic devices for video interviews. Exclusion criteria were age under 18 years, a BMI below 17.5 or above 40, ongoing participation in another group psychotherapy programme, an initiation of or change in psychiatric medication during the 8 weeks prior to the study, a scheduled inpatient stay during the intervention period, physical contraindications for bouldering, certain psychiatric conditions (e.g., psychosis), and acute suicidality.

### Instruments

#### State mindfulness scale for physical activity—German version (SMS-PA-G)

The SMS-PA was specifically developed to capture mindfulness during physical activity [19]. To create a German version of the SMS-PA, the English version was translated into German. An independent, professional translator then retranslated this first translation into English. We then thoroughly discussed the German translation’s wording with several researchers and adapted it in light of the retranslation.

The SMS-PA-G consists of 12 items, with the first six items focussing on mindfulness of the mind and the following six items focussing on mindfulness of the body. Ideally, the SMS-PA-G is administered directly after a physical activity and asks respondents to rate their experiences during said activity on a Likert scale ranging from 0 (*not at all*) to 4 (*very much*). In the original validation, positive correlations with state mindfulness (measured with the TMI) and state intrinsic motivation as well as negative correlations with state body surveillance (i.e., concerns about one’s looks during exercise) substantiated the construct validity [19]. Cronbach’s alpha was 0.90.

#### Multidimensional State Mindfulness Questionnaire (MSMQ) [33]

The MSMQ is a recently developed nine-item scale that measures state mindfulness. We used it in this study, as it is the only available state mindfulness scale validated in German. The MSMQ characterises mindfulness as a three-dimensional construct consisting of *Acting With Awareness*,* Nonjudgmental Acceptance*, and *Present-Moment Attention.* Each of the dimensions is captured with three items, of which the first six are reverse-coded. Respondents indicate on a scale ranging from 0 (*does not apply*) to 6 (*strongly applies*) the extent to which several statements (e.g., ‘I did things without paying attention’) apply to them. Total sum scores range from 0 to 54 with higher scores implying a larger degree of state mindfulness.

#### Skala Zur Allgemeinen Selbstwirksamkeit (SWE) [42]

The SWE is a 10-item scale that measures self-efficacy, which is the belief in one’s ability to manage difficult situations. Respondents rate statements on a 4-point scale ranging from 1 (*not true*) to 4 (*exactly true*). Total sum scores range from 10 to 40, with higher scores implying a stronger sense of self-efficacy.

#### Leipzig Short Scale (SOC-L9) [43, 44]

The SOC-L9 consists of nine items and is the short version of Antonovsky’s 29-item Sense of Coherence Scale (SOC-29). It captures respondents’ sense of coherence, which is a general and enduring feeling of trust that one’s inner and outer worlds are predictable and that circumstances will develop as may reasonably be expected. According to the model of Salutogenesis, such an attitude increases resilience and supports health and well-being.

On a 7-point scale ranging from 1 to 7, respondents place themselves between two contradictory labels that answer a question or end a statement (e.g., ‘In the future, you expect that your own life will (1) have no purpose at all| (7) be full of purpose’). Total sum scores range from 9 to 63, with higher scores indicating a stronger sense of coherence.

#### Generalized Anxiety Disorder Scale-7 (GAD-7) [45]

The GAD-7 is a short self-report questionnaire consisting of seven items. On a 4-point scale ranging from 0 (*not at all*) to 3 (*nearly every day*), respondents report how often they experienced the seven core symptoms of general anxiety disorder in the past 2 weeks. Total sum scores range from 0 to 21, and higher scores indicate stronger anxiety (minimal: 0–4; mild: 5–9; moderate: 10–14, severe: 15–21).

#### Montgomery-Asberg Depression Rating Scale (MADRS) [46]

The MARDS is a structured, clinician-rated interview form that measures respondents’ depression. It consists of 10 items that cover the core symptoms of depression: reported sadness, apparent sadness, inner tension, reduced sleep, reduced appetite, concentration difficulties, lassitude, inability to feel, pessimistic thoughts, and suicidal thoughts. Items yield a score ranging from 0 to 6. Total sum scores thus range from 0 to 60 with higher scores implying increased symptom severity.

#### Bewegungs- Und Sportaktivität Fragebogen (BSA-F) [47]

The BSA-F measures the extent to which respondents engage in physical activity. It divides physical activity into three parts representing activity at work, activity during leisure, and sports activity. The BSA-F’s items typically apply to a reference period of 4 weeks, which may be adapted to suit the purpose of the study. Activity at work is assessed on a 4-point scale ranging from 0 (*no activity*) to 3 (*a lot of activity*). Leisure activity and sports activity are measured by asking respondents for the type, frequency, and duration of activities in these areas. In addition, the sports part asks whether respondents were generally active over the previous 4 weeks. The scale’s responses may be compiled into a physical activity index.

#### Diagnostic and Statistical Manual of Mental disorders (DSM-V) diagnostic criteria [48]

We used the DSM-V diagnostic criteria to screen for depression. They consist of nine criteria, which may be divided into two main and seven specific depression symptoms. Via yes and no questions, participants indicate how many of the symptoms were present over the previous 2 weeks. If at least one main criterion and four side criteria are fulfilled, the screening is positive.

### Data recording

All data were recorded in the online platform RedCap2 [49]. Participants received a link via email and completed an online form containing the SMS-PA-G, the MSMQ, the SWE, the SOC-L9, and the GAD-7. The diagnosis of depression (DSM-V) and the MADRS external rating sum score were collected by staff in a video interview. All personnel involved in data gathering and handling were thoroughly trained.

### Sample

The validation of the SMS-PA-G used baseline (t0) data from the 129 participants enrolled in the ‘New ways to cope with depression’ trial (Table 1). The mean age was 39.8 years, and the proportion of women was 63%. A total of 71% of participants had received a Grammar school diploma. At the time of enrolment, participants suffered from an average of 1.7 main depression symptoms (out of 2) and 5.1 specific symptoms (out of 7), and 57% were taking psychiatric medication. The mean Body Mass Index (BMI) of 24.6 lay at the upper end of the normal range (18.5–24.9).

**Table 1:** Sample characteristics

Characteristics	*N* = 129
Age, *M* (*SD*)	39.8 (14.4)
Women, *n (%)*	81 (62.8)
Schooling* n (%)*	
Unfinished	2 (1.6)
Secondary School − 9 years	6 (4.7)
Vocational School − 10 years,	26 (20.2)
Grammar School − 13 years	91 (70.5)
BMI, *M* (*SD*)	24.6 (4.9)
Psychiatric Medication, *n (%)*	74 (57.4)
# Main symptoms of depression (out of 2), *M*	1.7
# Specific symptoms of depression (out of 7), *M* (*SD*)	5.1 (1.0)
SMS-PA-G (Range 0–48), *M* (*SD*)	25.3 (8.5)
SMS-PA-G Mind subscale (Range 0–24), *M (SD)*	12.4 (4.7)
SMS-PA-G Body subscale (Range 0–24), *M (SD)*	12.9 (5.4)

*Note* Schooling information was available for only 125 participants

*BMI* Body Mass Index, *SMS-PA-G* State Mindfulness for Physical Activity– German version

### Statistical analyses

We conducted a missing data evaluation. Through the expectation maximisation algorithm, missing values were imputed at the subscale level for two participants. Apart from the confirmatory factor analysis, all analyses were carried out in SPSS version 28.

#### Reliability and item analyses

We calculated Cronbach’s α for the SMS-PA-G and its *Mind* and *Body* subscales. Values above 0.7 were regarded as satisfactory [50]. In addition, we determined the discriminatory power of the items and the sensitivity of Cronbach’s α to the removal of single items from the scale.

#### Confirmatory factor analysis

We used the Lavaan package (version 0.6–15) to compute a confirmatory factor analysis in *R* [51]. Given the importance of testing several plausible rival models to identify the model that actually fits the data best [52], we investigated three models: a one factor model with a general mindfulness factor, a two-factor model with mind and body factors loading on six items each, and a bifactor model in which the variance in the indicators was explained by both mind and body factors and a general mindfulness factor. We considered a higher order factor model (general mindfulness loading on body and mind subfactors). However, such a second-order factor model with two subfactors was underidentified and thus required further constraints [53]. Once we imposed these constraints, the higher order model became widely equivalent to the two-factor model (same fit indices, same standardised factor loadings), did not provide additional insights, and was hence dropped from the analysis.

We used the WLSMV estimator to fit the models. This estimator is suitable for ordinal data and is robust to modest sample sizes [53, 54]. In addition, it was employed in previous validations of the SMS-PA [19, 22], thus enhancing comparability.

In scaling the latent variables, we used the variance standardisation method to provide standard errors and significance testing for all parameter estimates (as opposed to the marker method, where the first indicator is typically fixed to 1). However, this decision has no bearing on model fit or fully standardised factor loadings [53].

To assess model fit comprehensively, fit indices covering absolute fit, parsimony, and comparative fit ought to be reported [53]. Hence, we used the χ² significance test (cut-off: *p*-value > 0.001), the root mean square error of approximation (RMSEA; cut-off: < 0.06), the Comparative Fit Index (CFI, cut-off: > 0.95) and the Tucker-Lewis-Index (TLI, cut-off: > 0.95) to compare model fit (see [55] for cut-offs). In addition, we employed the weighted root mean square residual (WRMR, cut-off < 1.0) [56] because the standardised root mean square residual (SRMR) does not perform well with categorical data [53].

#### Construct validity

We computed Pearson correlation coefficients (*r*) to assess the hypothesised relationship between physical activity mindfulness and other related constructs. For the SMS-PA-G, we expected significant positive associations with the MSMQ sum score, the SWE, and the SOC-L9. Also, the SMS-PA-G was assumed to have a medium to strong relationship with the MSMQ subscales *Acting With Awareness* and *Attention* (as indicated by an *r* between 0.3 and 0.5 [57]) and a weak relationship (*r* <.2) with *Nonjudgment*. Conversely, we expected significantly negative correlations with the MARDS and the GAD-7.

#### Validity evidence via expected group mean differences

We used item 5 (this item asks whether the participant was regularly active over the previous 4 weeks**)** from the ‘Bewegungs- und Sportaktivität Fragebogen’ (BSA-F) [47] to divide participants into two groups: (1) engaging in regular physical activity and (2) not engaging in regular activity. We computed a *t* test to compare the levels of physical activity mindfulness in these groups. Given that physical activity mindfulness should differ between those who are regularly active and those who are not, we considered a significant difference to be supportive of the SMS-PA’s construct validity.

## Results

### Reliability and item analyses

For the sample in this study, the mean SMS-PA-G was 25.3 with a standard deviation of 8.5. Values ranged from 0 to 45 and thus spanned almost the entire range of the scale (0–48). The means of the single items ranged from 1.6 to 2.3. Cronbach’s alpha was 0.87 for the entire SMS-PA-G and 0.81 and 0.90 for the *Mind* and *Body* subscales, respectively. Apart from Item M3 (0.28), the discriminatory power of the items was rather high, ranging from 0.46 to 0.77. If single items were removed, Cronbach’s alpha remained steady between 0.85 and 0.88. Table 2 presents the item characteristics.

**Table 2:** Item translation with item characteristics of the SMS-PA-G

No	Original version item	Translation to German	*M*	*SD*	Discriminatory power	Cronbach’s alpha if item deleted
M1	I was aware of different emotions that arose in me.	Ich war mir verschiedener Emotionen bewusst, die in mir entstanden sind.	2.2	1.0	0.58	0.86
M2	I noticed pleasant and unpleasant emotions.	Ich habe angenehme und unangenehme Emotionen wahrgenommen.	2.3	1.1	0.46	0.87
M3	I noticed pleasant and unpleasant thoughts.	Ich habe angenehme und unangenehme Gedanken wahrgenommen.	2.3	1.1	0.28	0.88
M4	I noticed emotions come and go.	Ich habe wahrgenommen. wie Emotionen kamen und gingen.	2.0	1.1	0.56	0.86
M5	I noticed thoughts come and go.	Ich habe wahrgenommen. wie Gedanken kamen und gingen.	2.1	1.0	0.57	0.86
M6	It was interesting to see the patterns of my thinking.	Es war interessant. meine Denkmuster zu beobachten.	1.6	1.2	0.54	0.86
B1	I focused on the movement of my body.	Ich habe mich auf die Bewegungen meines Körpers konzentriert.	2.3	1.2	0.53	0.86
B2	I felt present in my body.	Ich habe mich präsent in meinem Körper gefühlt.	2.2	1.1	0.64	0.86
B3	I listened to what my body was telling me.	Ich habe auf das gehört. was mein Körper mir mitteilte.	2.0	1.1	0.64	0.86
B4	I was aware of how my body felt.	Ich war mir bewusst. wie mein Körper sich anfühlte.	2.3	1.1	0.77	0.85
B5	I noticed the sensations in my body.	Ich habe die Empfindungen in meinem Körper wahrgenommen.	2.2	1.0	0.71	0.85
B6	I was in tune with how hard my muscles were working.	Ich war im Kontakt damit. wie stark meine Muskeln arbeiteten.	1.9	1.2	0.51	0.87
Total			25.3	8.5		

*Note* Item characteristics based on participants for whom single item scores were available (*N* = 127)

*M* Mean, *SD* Standard Deviation, *M1-M6* Mind subscale, *B1-B6* Body subscale

### Confirmatory factor analysis

Table 3 shows the fit indices for the three models that were evaluated. For the one-factor model, none of the fit indices were in the acceptable range. For both the two-factor and bifactor models, the WRMR below 1 and the CFI and the TLI above 0.95 indicated good model fit. However, only the bifactor model had a nonsignificant Chi-Square test as well as an RMSEA below 0.06. Hence, the bifactor model with a general mindfulness factor and specific mind and body factors fit the data best. Table 4 contains the general factor loadings and the specific factor loadings for the bifactor solution. Apart from the general factor loading of M3 and the specific factor loading of M6, all standardised loadings were significant at the *p* <.05 significance level. The variance of each indicator explained by the factors (i.e., R^2^) fell between 47% and 79%.

**Table 3:** Confirmatory factor analysis fit indices

Model	Chi-square	*df*	*p*	RMSEA	CFI	TLI	WRMR
One factor	212.16	54	< 0.001	0.14	0.89	0.87	1.39
Two factors	107.60	53	< 0.001	0.08	0.96	0.96	0.91
Bifactor	55.79	42	0.075	0.04	0.99	0.99	0.49

*Note df* degrees of freedom, *RMSEA* root mean square error of approximation, *CFI* comparative fit index, *TLI* Tucker-Lewis index, *WRMR* weighted root mean square residual

**Table 4:** Confirmatory factor analysis results for bifactor model

No	Standardised general factor loadings	Standardised specific factor loadings	*R* ^2^
M1	0.59**	0.40**	0.50
M2	0.36*	0.66**	0.57
M3	0.11	0.85**	0.73
M4	0.58**	0.40**	0.49
M5	0.60**	0.35**	0.49
M6	0.68**	0.08	0.47
B1	0.35**	0.64**	0.54
B2	0.48**	0.72**	0.75
B3	0.56**	0.54**	0.60
B4	0.70**	0.55**	0.79
B5	0.68**	0.41**	0.63
B6	0.34**	0.61**	0.49

*p <.05. **p <.01

### Construct validity

Contrary to our assumption, physical activity mindfulness as measured with the SMS-PA-G was not significantly correlated with state mindfulness as measured with the MSMQ sum score (*r* =.11). Only the SMS-PA-G *Body* subscale showed a weak significant correlation of 0.19 with state mindfulness as measured by the MSMQ. On MSMQ subscale level, the *Acting With Awareness* subscale and the SMS-PA-G had a correlation coefficient close to zero (*r* =.05). Also, the *Nonjudgmental Acceptance* subscale showed no correlation (*r* = −.13) with the SMS-PA-G. In contrast, the *Present-Moment Attention* subscale did have a moderate correlation (*r* =.38) with physical activity mindfulness. In line with our assumption, positive cognitions as captured with the SWE (*r* =.37) and the SOC-L9 (*r* =.25) coincided with higher levels of physical activity mindfulness. Conversely, an inverse relationship between physical activity mindfulness and indicators of psychological burden (i.e., anxiety: *r* =.00; depression: *r* = −.17) could not be ascertained. Table 5 provides an overview of the correlations.

**Table 5:** Correlations between the SMS-PA-G and related constructs

Model	Mindfulness	Mind	Body	Range	Mean	SD
State mindfulness (MSMQ)	0.11	− 0.03	0.19*	0–54	22.03	9.09
Acting With Awareness subscale	0.05	− 0.04	0.11	0–18	8.12	4.58
Nonjudgmental Acceptance subscale	− 0.13	− 0.20*	− 0.04	0–18	6.55	4.28
Present-Moment Attention subscale	0.38**	0.22*	0.40**	0–18	7.36	3.48
Self-Efficacy (SWE)	0.37**	0.18*	0.42**	10–40	21.07	4.71
Sense of Coherence (SOC-L9)	0.25**	0.08	0.33**	9–63	28.45	7.73
Anxiety (GAD-7)	0.00	0.15	− 0.13	0–21	11.88	4.31
Depression (MARDS)	− 0.17	− 0.09	− 0.19*	0–60	27.29	7.41

*p <.05. **p <.01

### Validity evidence via expected group mean differences

The regularly active group significantly surpassed the nonactive group by 4.33 points (*p* =.002) on the SMS-PA-G (Table 6). The means of the *Mind* and *Body* subscales also differed significantly by 1.55 (*p* =.03) and 2.78 (*p* =.002) points, respectively.

**Table 6:** SMS-PA-G means and standard deviations for subgroups

Model	Mindfulness Mean (SD)	Mind Mean (SD)	Body Mean (SD)
Subgroup 1– No regular physical activity (*N* = 66)	23.21 (8.64)	11.68 (4.16)	11.53 (5.60)
Subgroup 2– Regular physical activity (*N* = 61)	27.54 (8.06)	13.23 (5.23)	14.31 (4.98)
Subgroup difference	4.33**	1.55*	2.78**

*Note* Participants for whom physical activity behaviour was known were compared (*N* = 127)

*p <.05. **p <.01

## Discussion

In this study, we used data from the ‘New ways to cope with depression’ trial to provide validity evidence for the German version of the State Mindfulness Scale for Physical Activity (SMS-PA-G) in a clinical sample. We produced sound evidence for the internal consistency and discriminatory power of the SMS-PA-G’s items. Also, we managed to replicate the bifactor structure of the SMS-PA found in the original validation study [19]. Construct validity was demonstrated, but contrary to our hypotheses, we did not find a negative correlation with anxiety and depression. Physically active participants yielded significantly higher SMS-PA-G values than nonactive participants.

The item analysis of the SMS-PA-G provided encouraging results. Cronbach’s alpha was in a satisfactory range for the total scale as well as the subscales. Also, Cronbach’s alpha fluctuated only minimally when single items were removed. The discriminatory power of the items was generally high. Only one item (M3) did not reach the cut-off (0.3) of medium discriminatory power [58]. In the English version, this item reads: ‘I noticed pleasant and unpleasant thoughts’. This statement is rather factual and might imply less of the neutrally hovering attention that is characteristic of mindfulness and is present in the other items to a larger degree, such as M4 (‘I noticed emotions come and go’). In a measure of mindfulness, M3 may have therefore elicited lower discriminatory power. It needs to be noted, though, that there were no irregularities regarding Item M3 in previous validations of the scale [19, 22].

A confirmatory factor analysis of the SMS-PA-G showed that the scale could best be described with a bifactor model. Bifactor models are best suited for unidimensional constructs with subdomains that carry some unique variance [53]. For the SMS-PA-G, we identified a general mindfulness factor as well as domain-specific mind and body factors. Hence, respondents’ general mindfulness levels will considerably determine their SMS-PA-G score. However, it will also play a role to what extent they are presently observant of the workings of their mind and body, respectively. This conceptualisation appears practical and is most in line with the theoretical groundwork laid by Tanay and Bernstein [19]. Conversely, the two-factor model of physical activity mindfulness, which also showed rather favourable fit indices, unduly implies a multidimensionality of physical activity mindfulness. However, body and mind are not two fully separate dimensions; rather, they are both important objects towards which mindfulness may be directed [34].

Contrary to other outcomes from this study, the correlations computed to assess construct validity differed to some extent from the hypothesised values. Suggested by obvious conceptual overlap and strong correlations between state mindfulness (as measured with the TMI) and the SMS-PA [19], a positive association was expected between state mindfulness (as measured with the MSMQ) and the SMS-PA-G. Surprisingly, the association turned out to be close to zero. This might have been due to different target time spans in the instructions of each scale. Whereas the SMS-PA-G asked respondents to think back to their last physical activity session, the MSMQ prompted respondents to report on their state mindfulness since waking up. Another possible explanation lies in the formulation of the scales’ items. The TMI contains items that are very much akin to the SMS-PA’s items. For example, ‘I was aware of my thoughts and feelings without overidentifying them’ from the TMI is similar to Items M4 and M5 from the SMS-PA. The MSMQ, on the other hand, does not contain such similarities. Its *Acting With Awareness* subscale focusses on awareness of things and activities, which are awareness foci other than ‘mind’ and ‘body’. Such a differential focus might have driven the low associations between the *Acting With Awareness* subscale and the SMS-PA-G and by extension the low associations between MSMQ total and SMS-PA-G total. The SMS-PA-G and the other MSMQ subscales were related as expected: the SMS-PA-G’s strong emphasis on *Present-Moment Attention* resulted in a positive correlation with the subscale, and its negligible emphasis on *Nonjudgmental Acceptance* resulted in a zero correlation. The latter was recently addressed by the development of the SMS-PA-2, which added acceptance items to the SMS-PA in order to provide a more complete representation of the physical activity mindfulness construct [36].

In line with earlier findings [8], we found the assumed association between indicators of self-ascribed competence and optimism (i.e., self-efficacy and sense of coherence) and physical activity mindfulness. As participants were confident in themselves and their capacities, they seemed to be better able to focus on the activity at hand. The association with indicators of emotional burden (i.e., anxiety and depression) found in previous mindfulness scale validations [29, 31] did not materialise as we had expected. One reason for this lack of finding may be that this study’s sample consisted of clinically depressed participants. There might not have been sufficient variance in mood that could vary with the SMS-PA-G and result in a discernible correlation. This idea is supported by the fact that, at 25.3, the mean SMS-PA-G score in this study was far lower than the mean SMS-PA score of 31.5 in the healthy sample from the original validation study [19]; this finding points to an association between physical activity mindfulness and negative shifts in mood.

Finally, we assessed the mean SMS-PA-G scores of active and nonactive participants. As those who regularly engage in physical activity should be more in tune with their minds and bodies, we expected the SMS-PA-G to register a gradient between the two groups. The substantially higher SMS-PA-G scores for the active group were supportive of the scale’s validity and paralleled SMS-PA results in the Spanish validation study [22].

At the outset, we described the critical relationship between mindfulness and physical activity habits. In sum, our results now indicate that physical activity mindfulness is associated with higher levels of physical activity and self-efficacy. The former result supports the notion that physical activity mindfulness may drive increases in physical activity– potentially via the link of intrinsic motivation (as theoretically underpinned by SDT). The latter result suggests that physical activity mindfulness might also drive physical activity levels through its link with self-efficacy. Indeed, several studies have found an association between mindfulness and self-efficacy [59, 60] and even between mindfulness and exercise self-efficacy [61]. Also, the mutually reinforcing connection between self-efficacy and physical activity is well-established [62]. Given these relationships, measuring physical activity mindfulness may help explore a possible virtuous cycle between mindfulness, self-efficacy, and physical activity. It may furthermore help investigate the respective roles of intrinsic motivation and self-efficacy as we utilise mindfulness to increase physical activity levels.

### Strengths and limitations

This study is the first to establish a scale for physical activity mindfulness in the German language. The data used in this study were generated in a rigorously designed randomised controlled trial, and the study rests on a multifaceted approach to validation. At the same time, though, our study comes with some limitations. Firstly, it needs to be noted that we tested the SMS-PA-G in a sample of depressed patients. Whereas the use of this sample provides particularly sound validity evidence for the use of the SMS-PA-G in clinical trials, it can potentially limit the generalisability of the results to the wider population. Secondly, owing to the overarching research design of the ‘New ways to cope with depression’ trial [41], the SMS-PA-G was not administered directly after a sports session. Rather, participants were asked to think back to their last physical activity. Of course, such recollections are less reliable than an immediate response. Thirdly, this study might have benefitted from a larger sample. At *N* = 129, the study’s sample size is on the lower side of numbers common in scale validation and confirmatory factor analysis.

### Opportunities for future research

The shortcomings addressed above open ample avenues for future studies. Another validation in a larger sample from the general population would strengthen the SMS-PA-G’s validity. Here, it would be ideal to disseminate the SMS-PA-G to participants right after a session of physical activity. Also, future research may provide additional clarity on the SMS-PA-G’s place among constructs, in particular its relationship with state and trait mindfulness. While the SMS-PA is already being used to assess the relationship between physical activity mindfulness and various outcome measures across interventions such as yoga [63], treadmill walking [64], and bouldering [65], the SMS-PA-G now facilitates similar research in German-speaking countries.

## Conclusion

The German translation of the SMS-PA measures physical activity mindfulness with sound internal consistency and discriminatory power. Its bifactor structure implies that physical activity mindfulness is in part a general mindset and in part consciousness of specific bodily and mental events experienced during exercise. Validity evidence was generally supportive of the SMS-PA-G. It showed that the SMS-PA-G places present-moment attention at the centre of the mindfulness construct, whereas other scales place more emphasis on a nonjudgemental attitude.

## Data Availability

Anonymised data are available upon reasonable request. Data are stored on protected devices at the University Hospital Erlangen. The Ethics Committee approved the model consent forms. These are also available upon reasonable request. Requests should be made to Jan Wallner (corresponding author) or Katharina Luttenberger.

## Abbreviations

BMI: Body Mass Index
BSA-F: Bewegungs- und Sportaktivität Fragebogen
CFI: Comparative Fit Index
DSM-V: Diagnostic and Statistical Manual of Mental Disorders– Version V
FFMQ: Five Facet Mindfulness Questionnaire
GAD-7: Generalized Anxiety Disorder Scale-7
KIMS: Kentucky Inventory of Mindfulness Skills
M: Mean
MAAS: Mindful Attention Awareness Scale
MADRS: Montgomery-Asberg Depression Rating Scale
MSMQ: Multidimensional State Mindfulness Questionnaire
RMSEA: Root Mean Square Error of Approximation
SD: Standard Deviation
SDT: Self-determination theory
SMS: State Mindfulness Scale
SMS-PA: State Mindfulness Scale for Physical Activity
SMS-PA-G: State Mindfulness Scale for Physical Activity– German Version
SOC-L9: Sense of Coherence - Leipzig Short Scale
SRMR: Standardised Root Mean Square Residual
SWE: Skala zur Allgemeinen Selbstwirksamkeit
TLI: Tucker-Lewis-Index
TMI: Toronto Mindfulness Inventory
WLSMV: Weighted Least Square Mean and Variance Adjusted
WRMR: Weighted root mean square residual
